# All-printed highly sensitive 2D MoS_2_ based multi-reagent immunosensor for smartphone based point-of-care diagnosis

**DOI:** 10.1038/s41598-017-06265-1

**Published:** 2017-07-19

**Authors:** Memoon Sajid, Ahmed Osman, Ghayas Uddin Siddiqui, Hyun Bum Kim, Soo Wan Kim, Jeong Bum Ko, Yoon Kyu Lim, Kyung Hyun Choi

**Affiliations:** 1 Department of Mechatronics Engineering, Jeju National University, Jeju, Korea; 2 Faculty of Advanced Convergence Technology and Science, Jeju National University, Jeju, Korea; 3 Biotechnology Research Center, Suez Canal University, Ismailia, Egypt; 4 College of Veterinary Medicine, Jeju National University, Jeju, Korea

## Abstract

Immunosensors are used to detect the presence of certain bio-reagents mostly targeted at the diagnosis of a condition or a disease. Here, a general purpose electrical immunosensor has been fabricated for the quantitative detection of multiple bio-reagents through the formation of an antibody-antigen pair. The sensors were fabricated using all printing approaches. 2D transition metal dichalcogenide (TMDC) MoS_2_ thin film was deposited using Electrohydrodynamic atomization (EHDA) on top of an interdigitated transducer (IDT) electrode fabricated by reverse offset printing. The sensors were then treated with three different types of antibodies that were immobilized by physisorption into the highly porous multi-layered structure of MoS_2_ active layer. BSA was used as blocking agent to prevent non-specific absorption (NSA). The sensors were then employed for the targeted detection of the specific antigens including prostate specific antigen (PSA), mouse immunoglobulin-G (IgG), and nuclear factor kappa-light-chain-enhancer of activated B cells (NF-κB). IgG was then selected to test the sensors for point of care (POC) diagnosis through a specially designed electronic readout system for sensors and interfacing it with a smartphone using Bluetooth connection. The sensors showed promising performance in terms of stability, specificity, repeatability, sensitivity, limit of detection (LoD), and range of detection (RoD).

## Introduction

Immunosensors are compact analytical devices that are used to detect the formation of a complex resulting from an antigen-antibody interaction using transduction techniques to generate an interpretable and process able output signal. The different types of transducing mechanisms are based on difference in generated signals or changes in properties when a complex is formed^1^. Immunosensors are well recognized standard bio detection devices employed in laboratories for disease diagnosis, food industry for safety testing, and for general environmental contamination monitoring. The essential foundation of all the immunosensors is to specifically recognize antigens by antibodies to form a stable complex^2^. Immunosensors have been successfully fabricated and tested to reliably sense various bioactive elements like DNA^3^, viruses^4, 5^, enzymes and cell receptors^6^, glucose and other chemicals, proteins, and hormones^7, 8^, etc.

There are a number of working principles on which the immunosensors can be fabricated. They can be based on chromatography, fluorescence, electrochemical variation, mass spectrometry, surface plasmon resonance, lateral flow, etc. refs 9–11. Electrochemical immunosensors have received considerable attention owing to their ease of use, reasonable limit of detection (LoD) with a small sample volume, and being a simple analytical platform^12^. The most promising applications of electrical biosensors include situations where low cost, small setup size, and speedy results are crucial but high end and high accuracy results are not a priority for example, in point of care diagnosis for house hold personal use, emergency situations like in an ambulance, routine clinical checkups, water quality check, and screening^13^. There are further different categories in the electrochemical immunosensors based on their working principles that include coated paper based chemristors^14^, potentiometric sensors^15^, amperometric sensors^16^, sandwich type structures^17^, IDT based sensors^18^, and most commonly, field effect transistors (FETs)^19^.

FET based biosensors have attracted much attention owing to their rapid, inexpensive, and label-free detection. They have lower sensitivity as compared to other non-electrical methods but that problem is being addressed by using 1D and 2D structures like nanowires and 2D transition metal dichalcogenides (TMDCs) instead of bulk materials^20, 21^. TMDCs have multiple layers with strong in-plane bonds and weak out-of-plane interactions enabling easy exfoliation into 2D sheets having scalable bandgaps making them ideal for using in electronic sensing devices. The examples of these materials used for bio sensing include MoS_2_, MoSe_2_, WS_2_, and WSe_2_^20, 22, 23^. The most common among these TMDCs is MoS_2_ that has been employed in various sensing applications like gas sensing, chemical vapor sensing, and bio sensing, etc. based on its excellent electrical properties and comparatively easier synthesis and processing^24, 25^. Yet, the biggest limitations with using lower dimensional materials in FET based bio sensors are the severe fabrication challenges, impairing their practical applications^21^.

In this research work, we have fabricated an impedance based immunosensor using an interdigitated transducer (IDT) electrodes with MoS_2_ as the electrically active sensing layer. This design compensates for the lower sensitivity issues associated with general FET based sensors by offering a large surface area for detection and combines the advantages of TMDCs by implying a few-layered TMDC material as the active layer^26^. It further solves the commercialization problem as the fabrication techniques used are all printed, cheap, simple, highly accurate, and mass production compatible. The fabricated sensor is aimed for clinical commercialization to detect general antibody-antigen based bioactive elements. Three different biomarkers have been tested to analyze the bio sensing performance of the fabricated sensors. The results show promising performance making them suitable for applications to detect general bioactive elements for point-of-care (POC) diagnosis well within the required clinical ranges.

## Results

### Antibody immobilization and antigen detection protocol

A standard protocol with few modifications was adopted for immobilization of the antibodies. The antibodies were first diluted in saline/PBS to make solutions of 1 µg/ml protein concentration. 3% BSA was added to the antibody solutions for blocking and minimize the non-specific absorption (NSA)^27^. The fabricated sensors were then submerged into these solutions and were left for 1 hour on mechanical shaker for incubation. As a result, the antibodies/receptors were thoroughly physisorbed into the MoS_2_ sensing layer with the blank regions blocked by the BSA. The functionalized surface enables covalent bonding of the antibodies in addition to just flat dispersion on surface that is significant to improve the specificity. Further details about functionalization are presented in supplementary data. The sensors were rinsed with DI water to remove any traces of non-immobilized molecules.

The immobilization and specific binding was investigated by laser fluorescence microscopy. The as used NF-κB and IgG give red colored fluorescence for the antigens and green for antibodies. Glass slabs without electrodes coated with MoS_2_ were incubated in 1 µg/ml antibody solution for NF-κB and 10 µg/ml for IgG. Similar treatment was used for blocking and immobilization. The samples were then treated with three different concentrations of IgG antigen and a single concentration of NF-κB antigen. The results from the laser confocal microscopy were used to explain immobilization and specificity.

For electrical characterizations, a 100 µl control solvent was added to the active portion of the fabricated sensor and the impedance and AC effective series resistance (ESR) were recorded at 0% antigen concentration as the reference/control value. The followed protocol is similar to the water gated FET based sensors^21^ but the third (reference) electrode is not required in this scheme as CV is not used here but the net change in resistance between the two electrodes is recorded. A small sinusoidal voltage of a particular frequency was applied and the change in resulting AC current output was measured similar to chronoamperometry. The ratio of input voltage and output current gives the effective impedance. This method is also referred to as electrical impedance spectroscopy by some researchers^13^. The principle of impedance change is majorly dependent on dielectric charge transfer and electrolyte conductivity rather than the redox reactions. The advantage of using this scheme is that even if there is a redox reaction occurring in the faradic zone, the direct electron transfer will also result in the change in impedance of the sensor, thus improving the sensitivity by a great extent. Small amounts of specific antigens were added to the solvent that were selectively bound to the antibodies immobilized on MoS_2_ thin film resulting in impedance change. The real time data was automatically logged in and plotted on a computer with a sampling rate of 1 s. The complete process of detection and characterization is presented in the last figure in materials and methods portion. The concentration of the antigens were kept being increased to a point until the readings start to saturate and there was no significant change in response upon further increase in concentration.

### Point of care diagnosis

Point of care (POC) diagnosis is the real time sensing of the target analytes or bio-reagents. POC devices currently available for clinical tests include detectors of bacterial infections, viral infections, non-communicable diseases (PSA for prostate cancer, proteins for inflammation), and parasitic infections^28^. In this research work, we selected IgG as the target analyte to be detected using the POC setup. The same system can be used with other bio-reagents after individual calibration. The system was first optimized using known concentrations of IgG and the trends were calculated. The POC system and step by step detection process is presented in Supplementary Figure S2. After calibration, the system was used for the quantitative detection of the IgG antigen. A smartphone application was designed for a guided seamless diagnosis. The readings are displayed after an average of 40 seconds when the system calculations are finished. The Supplementary Video-S1 shows the real-time measurement for a 400 ng/ml IgG sample.

### Physical and chemical properties

**Figure 1:**
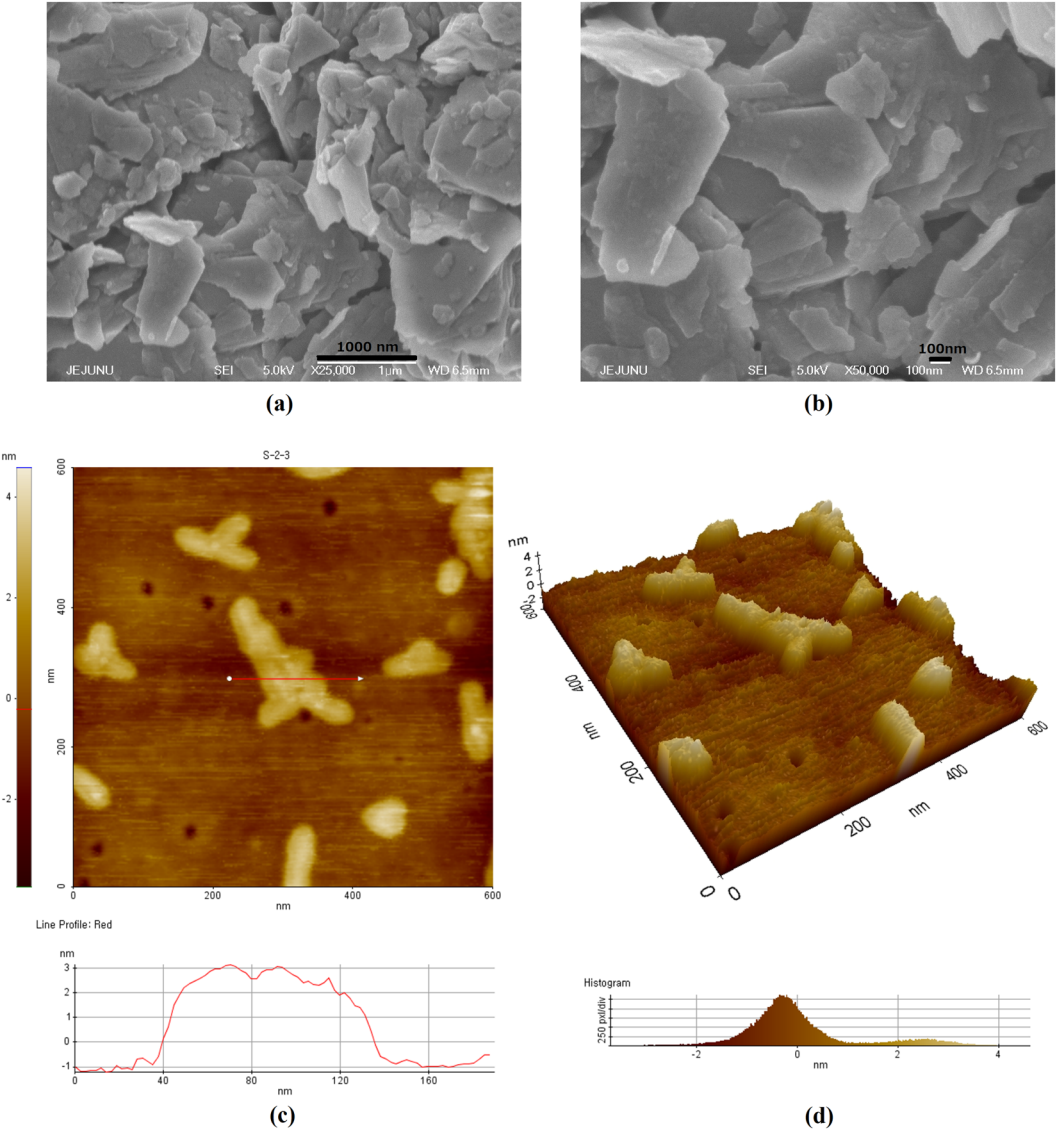
SEM and AFM of the active layer showing. (**a**) MoS_2_ active layer with highly porous surface, (**b**) 2D MoS_2_ flakes, (**c**) 2D surface profile and AFM line profile, and (**d**) 3D surface profile by AFM.

Figure 1(a,b) presents the SEM images of MoS_2_ thin film at different magnifications showing clear 2D sheets of the material and a highly porous active layer ideal for physisorption and bonding of antibodies on to the film. This facilitates direct electron transfer from the antibody-antigen complex to the electrodes for higher sensitivity^29^. The higher surface roughness also reduces the non-specific absorption of proteins^30^. Figure 1(c,d) shows the AFM images of the MoS_2_ flakes indicating few nanometer thick flakes ascertaining the exfoliation of the bulk into few layered sheets.

**Figure 2:**
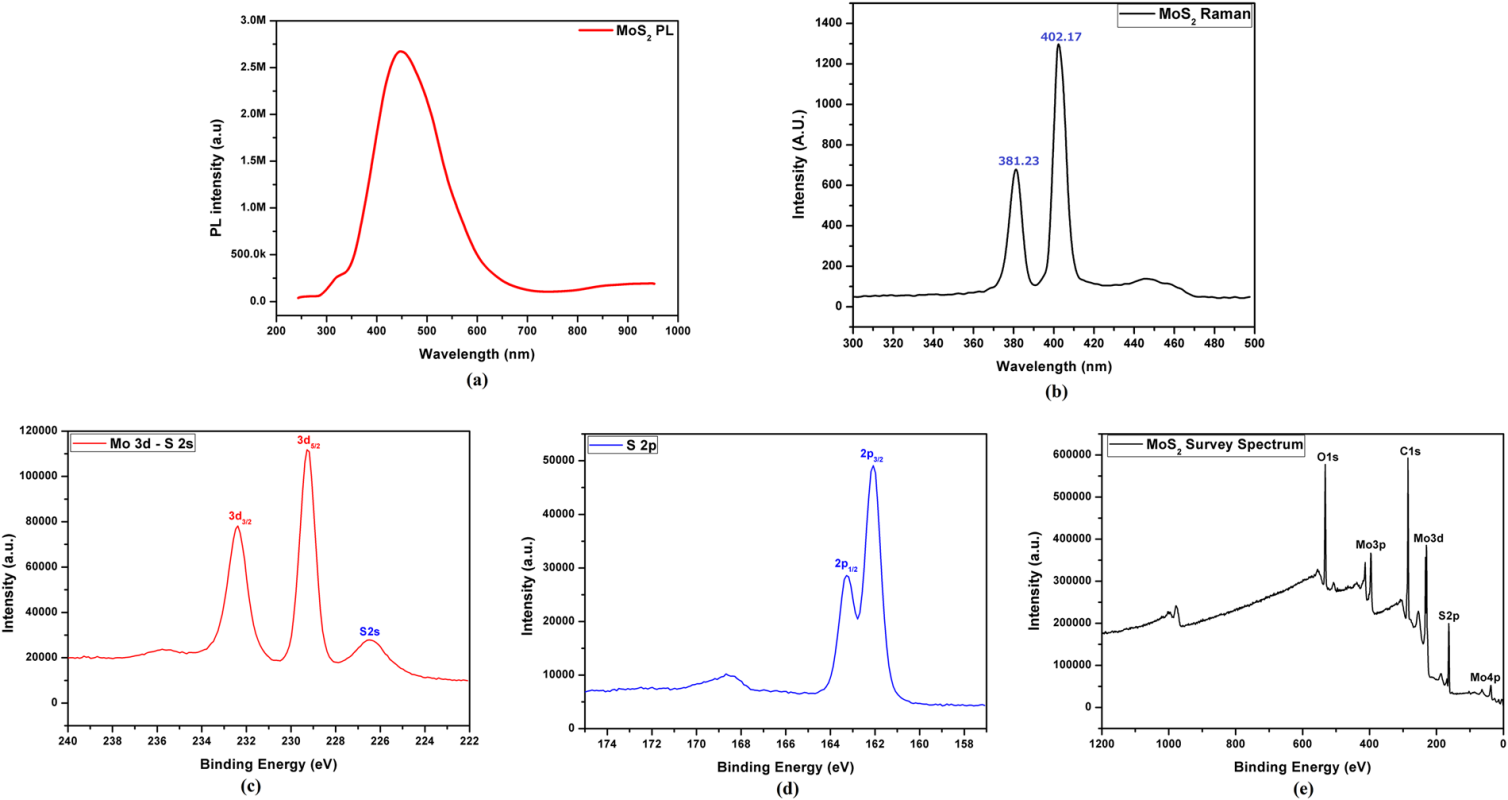
Chemical characterizations including. (**a**)PL emission spectrum, (**b**) Raman spectrum, (**c–e**) XPS spectra of MoS_2_ active layer.

Figure 2(a) shows the PL emission spectrum of MoS_2_ flakes with a broad peak at 440 nm. Usually this broad peak appears at 652 nm in case of large monolayer sheets exfoliated by scotch tape method but in this case, the shift in peak is due to direct and indirect bandgap in monolayer and few layer flakes respectively. This also might be due to mechanical grinding resulting in smaller flakes and possibly few quantum dots giving rise to quantum confinement effects^31^. The Raman spectrum results presented in Fig. 2(b) shows that the phonon modes of exfoliated MoS_2_ flakes lie at 381.23 nm and 402.17 nm ascertaining the attributes of Raman analysis for exfoliated MoS_2_ flakes^32^. The XPS results presented in Fig. 2(c–e) confirm the presence of only MoS_2_ as the active layer material with no contamination as such except the Carbon and Oxygen from the atmosphere. The atomic ratio of the thin film was 1:2 for Mo:S that ascertains the presence of MoS_2_ flakes.

**Figure 3:**
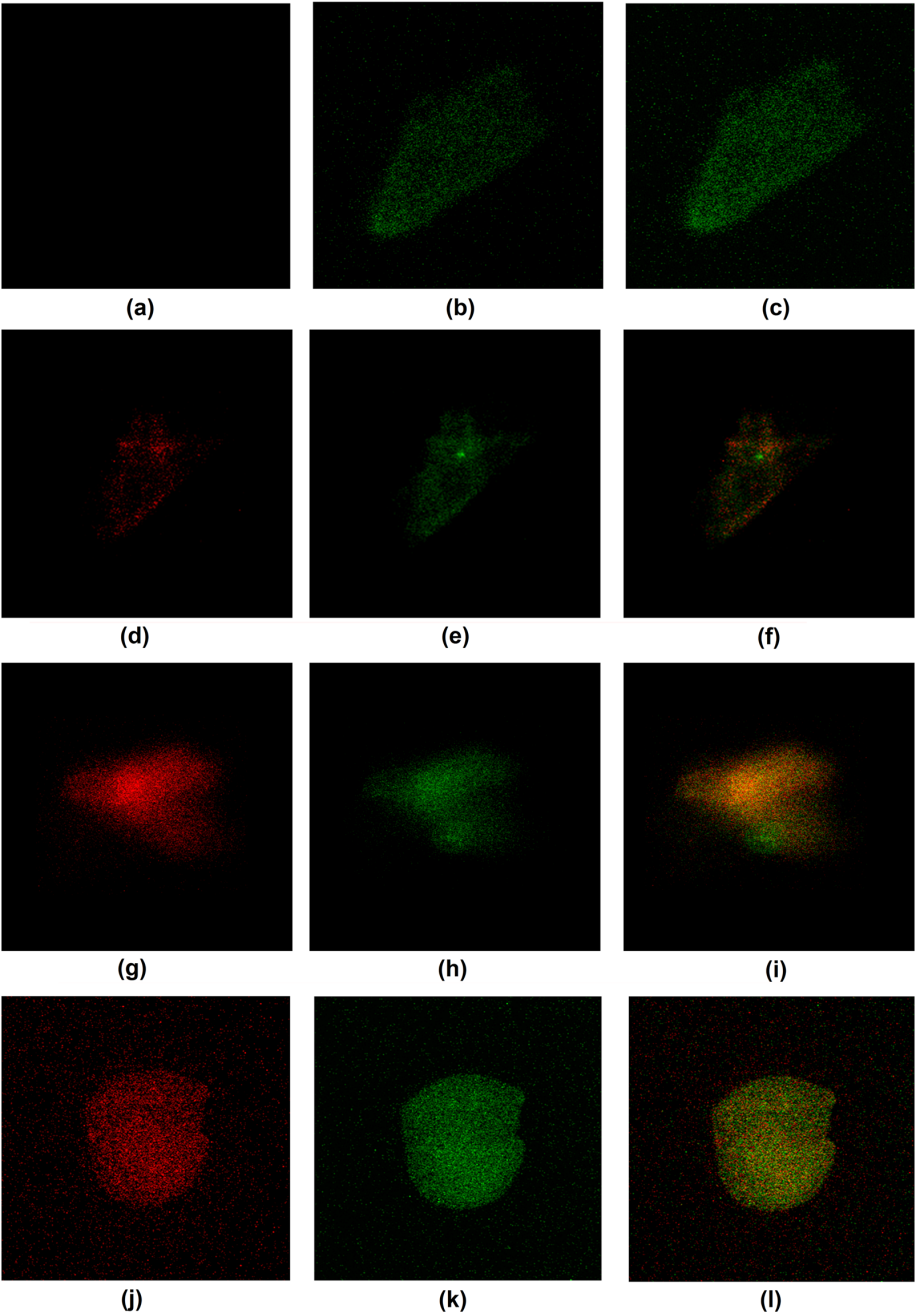
Confocal microscopic images showing antibody-antigen complex formation (**a**–**c**) zero IgG antigen concentration with only antibody visible (green), (**d**–**f**) 3 µg/ml IgG antigen (red) and IgG antibody (green), complex (orange), (**g**–**i**) 6 µg/ml IgG antigen (red), antibody (green), complex (orange), (**j–l**) NF-κB 400 ng/ml antigen (red), NF-κB antibody (green), complex (orange).

The laser confocal microscopy gives fluorescence only at the locations of immobilization for both antibodies and antigens. This process makes sure that only immobilized antibodies and bound antigens play their role in sensing behavior of the devices. No green fluorescence should be observed on the areas that are blocked and do not have immobilized antibodies. Similarly, no red fluorescence should be visible outside that area to confirm specific binding and selectivity of the sensor. The results presented in Fig. 3 show no antigen (red) fluorescence for control sample while both red and green colors are observable for different concentrations of antigens.

The color intensity for higher antigen concentrations is higher and the overlapping orange color intensities also increase in this case representing successful complex formation. Also, there is no fluorescence observable at surrounding areas of the immobilized antibody-antigen complex confirming the selective binding and specificity of the sensors.

### Detection of PSA

**Figure 4:**
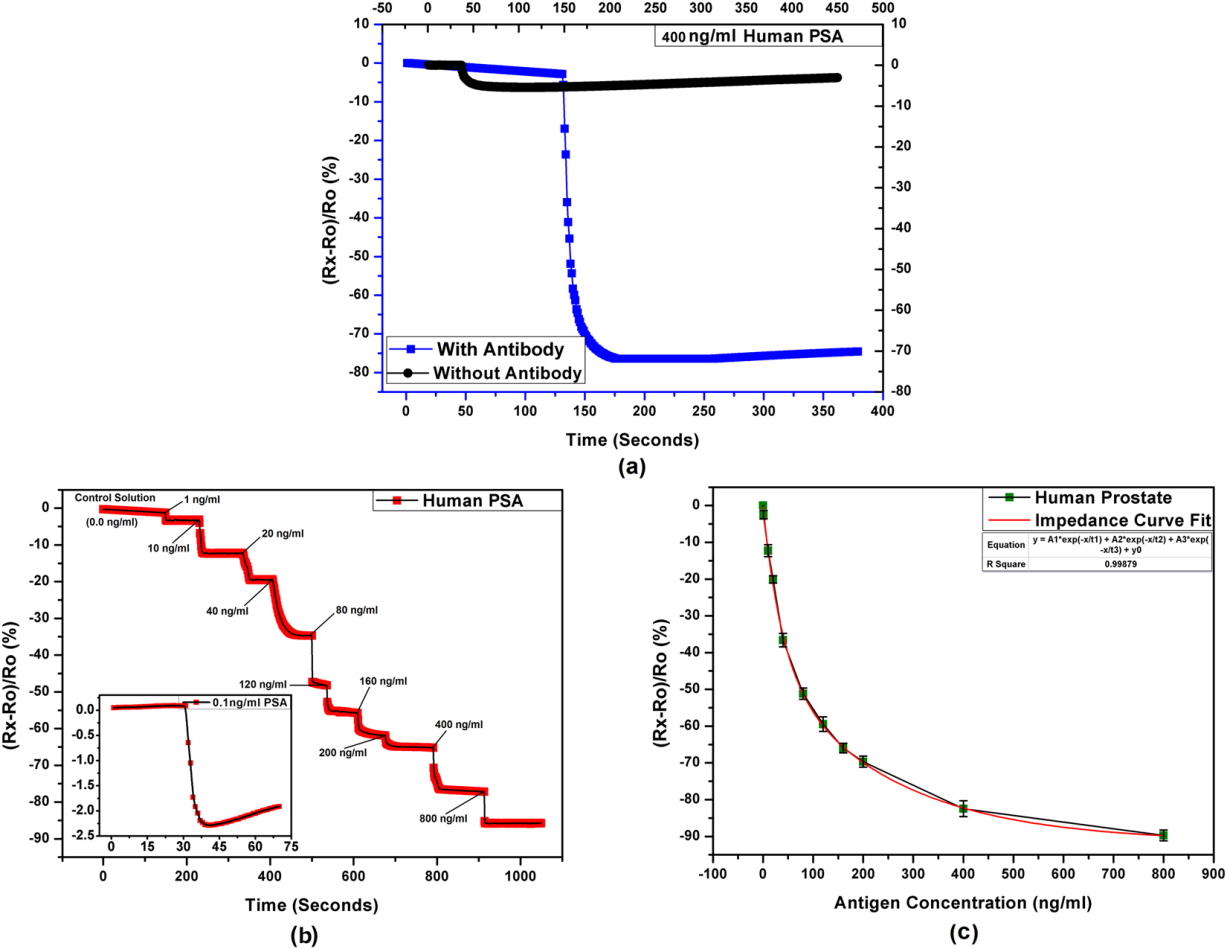
Output characteristics of PSA immunosensor (**a**) response for 400 ng/ml PSA with and without immobilized anti-PSA antibodies, (**b**) step response curve showing stable readings with time for increasing concentrations of PSA with inset showing LoD curve for 0.1 ng/ml PSA, and (**c**) response curve representing the behavior of the sensors for the full tested range of PSA concentrations.

PSA is a widely used clinical tumor biomarker for prostate cancer detection^33^. PSA sensors have a wide range of reported limits and ranges of detection including^33–35^. The minimum required detectable amount of PSA in human blood serum is 2 ng/ml for clinical diagnosi^13, 36^ while the normal range is 0 to 4 ng/ml. Figure 4 presents the response of the fabricated sensors for quantitative detection of PSA. The results indicate that the immunosensors are highly responsive towards PSA detection.

In Fig. 4(a), the effect of target specific detection through antibody immobilization is investigated. The sensors coated with MoS_2_ thin film with and without immobilized antibodies were used for PSA detection. It can be seen that the resistance of the sensor still drops a little bit for a 400 ng/ml PSA solution due to the general conductivity of the protein and sudden distortion in the electric field of the IDT but as there are no antibody attachment sites available on the surface, the effect slowly dies off and the resistance slowly climbs back up. It is important to note that the maximum change is only ~8% in this case that further reduces to ~3% after few minutes. The same concentration of PSA for the sensor with immobilized antibodies shows a ~78% change in impedance and the readings remain stable even after 6 minutes indicating the successful antibody-antigen complex formation. This shows a signal to noise ratio (SNR) of >10 that has a high significance value^37^. These results confirm high selectivity of the sensors for the specific type of antigen detection with negligible response towards unwanted compounds present in the test sample.

Figure 4(b) shows the step response of the sensors at increasing concentration of PSA in the solution. The time based response indicates quick detection of varying concentrations of antigen in the solution and show that the readings remain stable. The concentration vs percentage change for three trials is presented in Fig. 4(c) that indicates the response curve shape of the sensor output towards different PSA concentrations. If the sensor is aimed for quantitative detection of the analyte, the range of detection (RoD) is very important. RoD is the ratio of the largest measurable target concentration and the limit of detection where the upper limit is set by the saturation of electrode/probe. The RoD for PSA for the fabricated sensor is 1 ng/ml to 800 ng/ml while the limit of detection is 0.1 ng/ml Fig. 4(b) inset. Both these values lie well within the required clinical ranges for prostate cancer diagnosis.

### Detection of IgG

**Figure 5:**
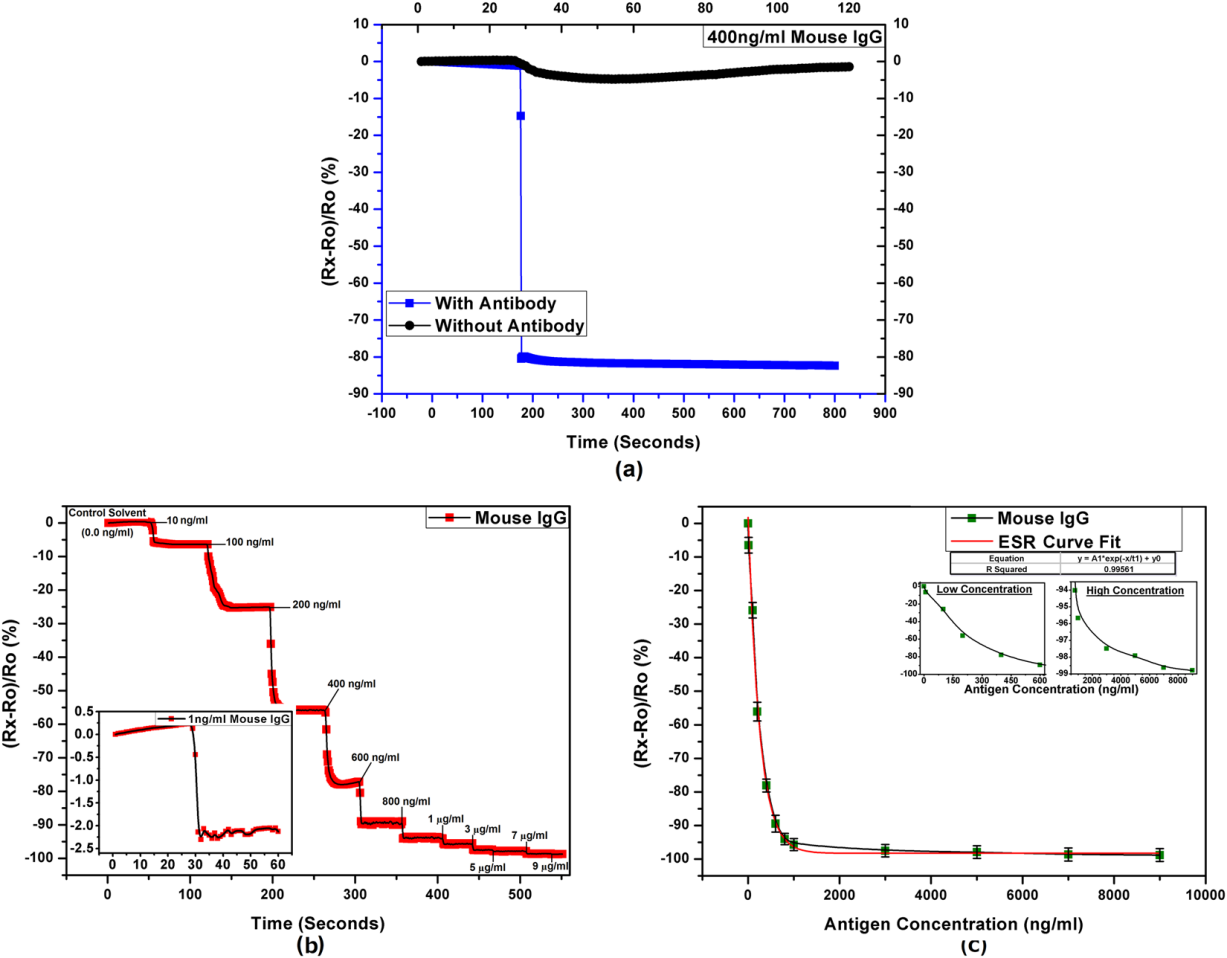
Electrical characteristics of the IgG immunosensor (**a**) response towards 400 ng/ml IgG with and without immobilized anti-IgG antibodies, (**b**) step response for increasing concentrations of IgG with inset showing LoD curve for 1 ng/ml IgG, and (**c**) response curve for the full tested range of IgG with insets showing the separated curves for low (0–800 ng/ml) and high (800–9000 ng/ml) concentration ranges.

Serum immunoglobulin level measurement is a routine clinical practice as it gives important information about humoral immune status^38^. Various sensors for the detection of IgG have been fabricated using a variety of sensing techniques and different ranges of LoD and RoD^17, 39–44^. Figure 5 presents the response of the fabricated sensors for quantitative detection of mouse IgG.

The results for 400 ng/ml IgG show a high significant SNR of >11 proving the high specificity of the sensors similar as in case of PSA. The LoD for IgG was found to be 1 ng/ml while the RoD of 9000 (1 ng/ml to 9 µg/ml) was achieved. The readings become quickly saturated for higher concentrations of IgG that was found to be the result of lower concentration of immobilized antibodies. For a better detection range, comparable amount of antibodies to the targeted amount of antigen have to be immobilized. In this case, the immobilized antibodies are in concentration of 1 µg/ml while the targeted detection is for 9 µg/ml of antigen. The results would have been much better if a 10 µg/ml solution was used for antibody immobilization as presented in case of POC diagnosis.

### Detection of NF-κB

**Figure 6:**
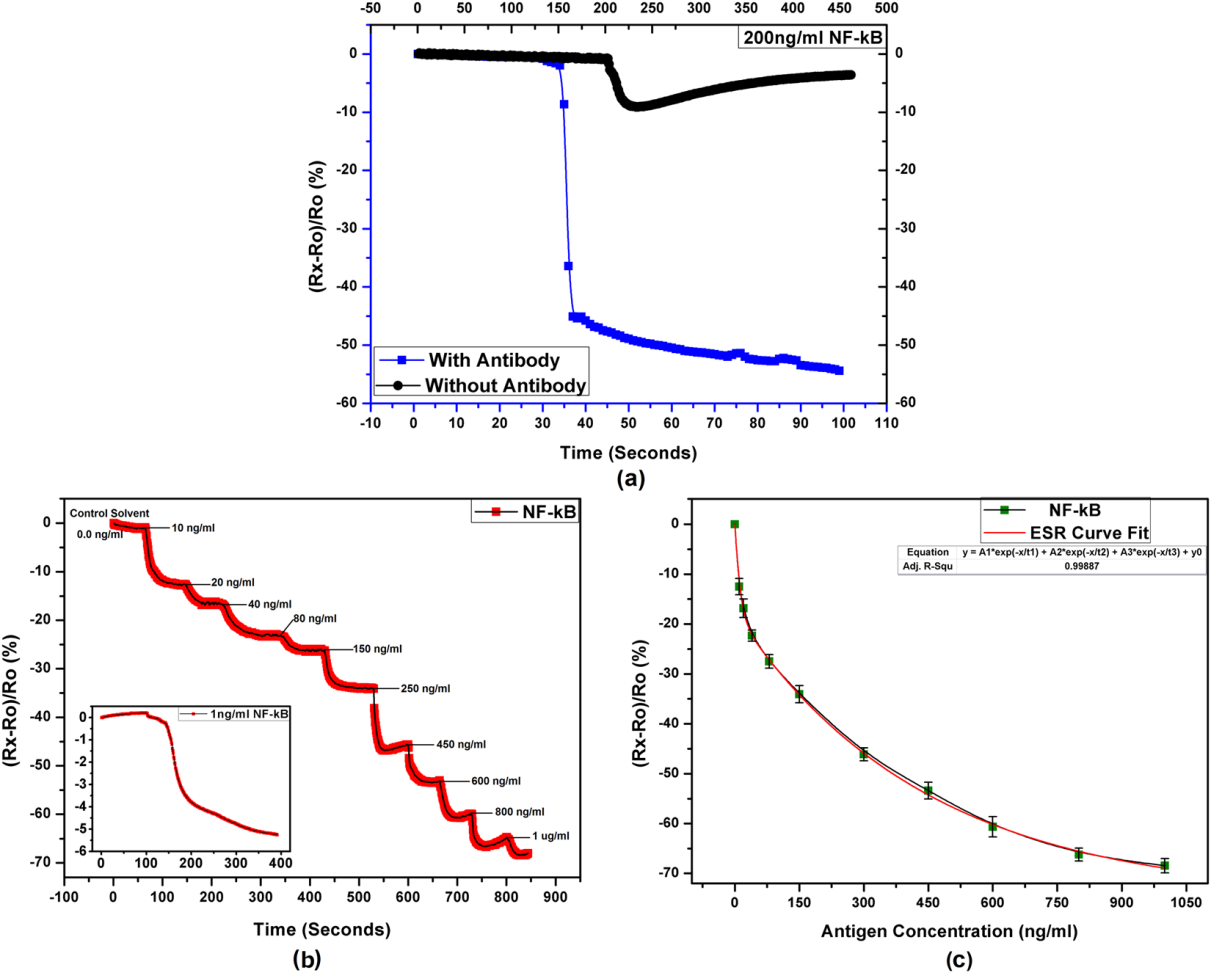
NF-κB immunosensor response (**a**) addition of 200 ng/ml NF-κB to sensors with and without immobilized antibodies, (**b**) step response showing stable readings with time for increasing concentrations of NF-κB with inset showing LoD curve for 1 ng/ml NF-κB, and (**c**) response curve for the full tested range.

Transcription factor of the nuclear factor κB (NF-κB) is involved in large number of genes regulation and is associated with diseases like inflammation, asthma, atherosclerosis, septic shock, arthritis, and even cancer^45, 46^. The antibody used here, NF-κB p65 recognizes endogenous levels of total NF-κB p65/RelA protein and does not cross react with other NF-κB/Rel family members. One of the few reported ones use biomarkers and optical methods and their LoD and RoD are not clearly defined^47^. The literature suggests that there is not enough work done on direct NF-κB quantitative detection. Figure 6 presents the response of the fabricated sensors for quantitative detection of NF-κB.

The results show that the SNR for NF-κB is ~5, the LoD is 1 ng/ml, while the RoD is 1000 (1 ng/ml to 1000 ng/ml).

### Repeatability and point of care diagnosis

**Figure 7:**
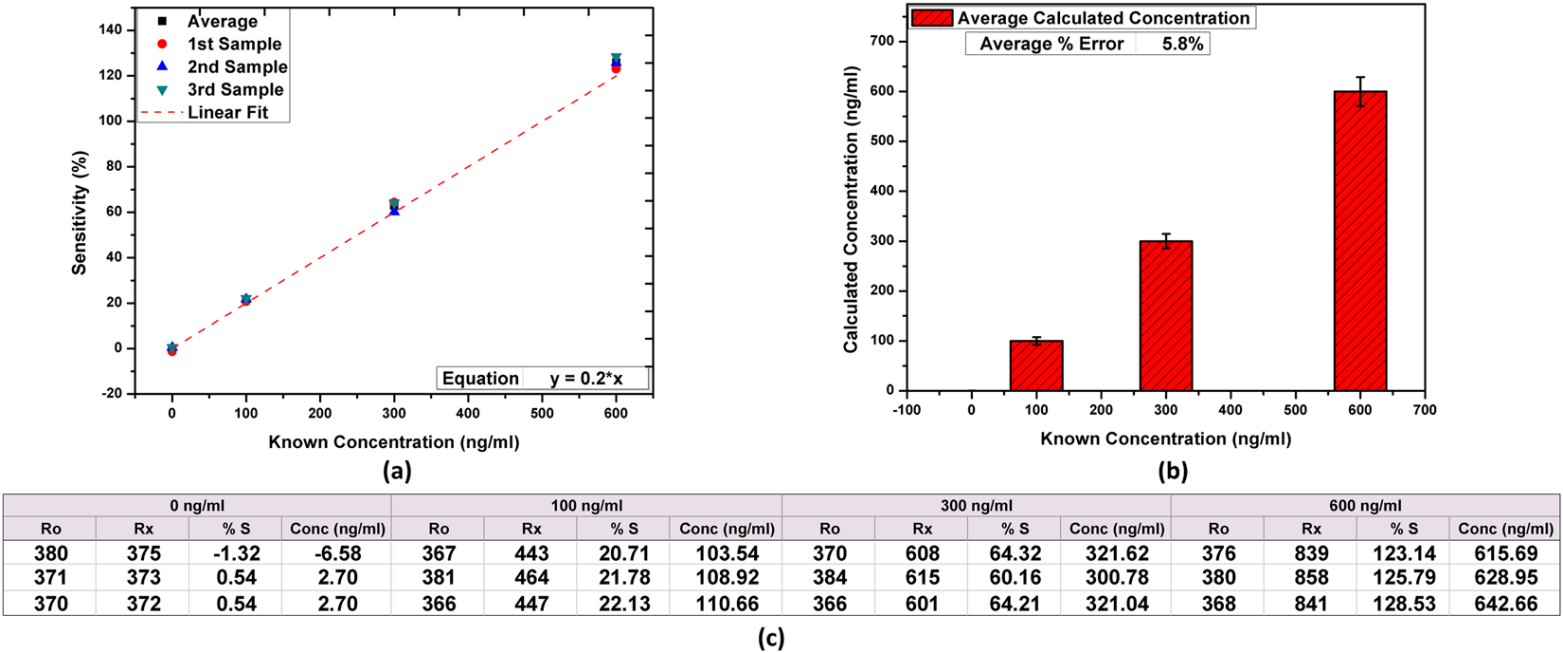
Results of IgG detection using POC setup (**a**) calibration readings for known concentrations, (**b**) difference between calculated and known values, and (**c**) presenting the table with all actual recorded readings from POC diagnosis setup.

The general purpose immunosensors were tested for POC diagnosis using IgG as the target bio-reagent. Four different concentrations of IgG were used for calibration of the test setup. Intensive programming routines were used for minimizing the error and improve repeatability. The system was optimized to detect the IgG in range of 0 ng/ml to 600 ng/ml where it shows almost linear behavior. The results of percentage sensitivity from the calibration readings for known solutions of 0 ng/ml (control), 100 ng/ml, 300 ng/ml, and 600 ng/ml having 3 sets of samples for each concentration are presented in Fig. 7. The results show stable behavior of the fabricated test setup with an average error of approximately ±5.8%.

For the final device evaluation, two different concentrations that were not used in calibration were detected using the setup. For 400 ng/ml solution, the output given by the system was 421 ng/ml that has an error of approximately +5.4%. For 200 ng/ml known concentration, the recorded output was 213 ng/ml showing +6.9% error. Further calibration and fine optimization can be done to make the results more consistent and further reduce the error percentage. For initial stages, the stability and repeatability of the presented diagnostic setup is very acceptable.

## Discussion

A general purpose immunosensor has been fabricated for quantitative detection of multiple bio-reagents with excellent stability and high sensitivity. A point of care diagnosis setup linked to smart phone through Bluetooth was also fabricated and an Android application was developed for end user interface. The detection protocol uses antibody-antigen complex formation and records the resulting change in impedance. Interdigitated type truly identical silver electrodes were fabricated using reverse offset printing technique and the active layer of exfoliated MoS_2_ flakes was deposited on top using Electrohydrodynamic atomization technique. The specific antibodies were immobilized into the porous semiconducting active layer through physisorption with BSA was used as blocking agent to avoid NSA. The use of 2D TMD material as the active layer enhances the sensitivity of the device by enabling operation in both faradic region (direct electron transfer for faradic current) and dielectric and ionic currents for impedance change. The detection limits of 0.1 ng/ml, 1 ng/ml, and 1 ng/ml were achieved for PSA, IgG, and NF-κB respectively. The detection ranges of 1 ng/ml to 800 ng/ml, 10 ng/ml to 9 µg/ml, and 10 ng/ml to 1 µg/ml were achieved for PSA, IgG, and NF-κB respectively. SNR values of 10, 11, and 5 were achieved for PSA, IgG, and NF-κB respectively showing high significance of the results. The POC diagnosis setup successfully detected the test samples of different concentrations of IgG with an average error of ±5.8%. The developed setup is ideal for commercialization as a general purpose household self-diagnostic kit after further refining using microfluidic chambers, higher resolution reference sensors, rigorous calibration using large number of samples and testing on various bio-reagents.

## Methods

### Materials and reagents

The anti-Prostate-specific Antigen antibody and the antigen were purchased from Sigma. The antibody had a protein concentration of ~1 mg/ml while the antigen solution had ~0.2–0.6 mg/ml protein content. Anti-Mouse IgG antibody buffered aqueous solution with concentration of 10–20 mg/ml and normal mouse IgG antigen with a concentration of 1 mg/ml were purchased from Sigma. NF-κB p65 rabbit mAb antibody with 100 µg/ml BSA solution was purchased from Cell Signaling Technology. NF-κB (p65) control peptide human antigen with ~1 mg/ml protein concentration was purchased from Sigma.

MoS_2_ ultrafine crystalline powder (average particle size: ~90 nm, Mol. Wt. 160.07, purity 99.0%) was purchased from Graphene Supermarket. N-Methyl-2-pyrrolidone (NMP) solvent to prepare MoS_2_ ink was purchased from Sigma. 30% Bovine serum albumin (BSA) solution in sodium chloride for blocking NSA was purchased from Sigma. 96% Butyltrichlorosilane (BTS) surfactant for functionalization was purchased form Sigma. For the fabrication of electrodes, silver conductive ink was used by Silverjet DGH ink for reverse offset (viscosity: 1.5 cps, surface tension: 24.4 mN/m, dispersion matrix: octane based).

### Electrode fabrication

**Figure 8:**
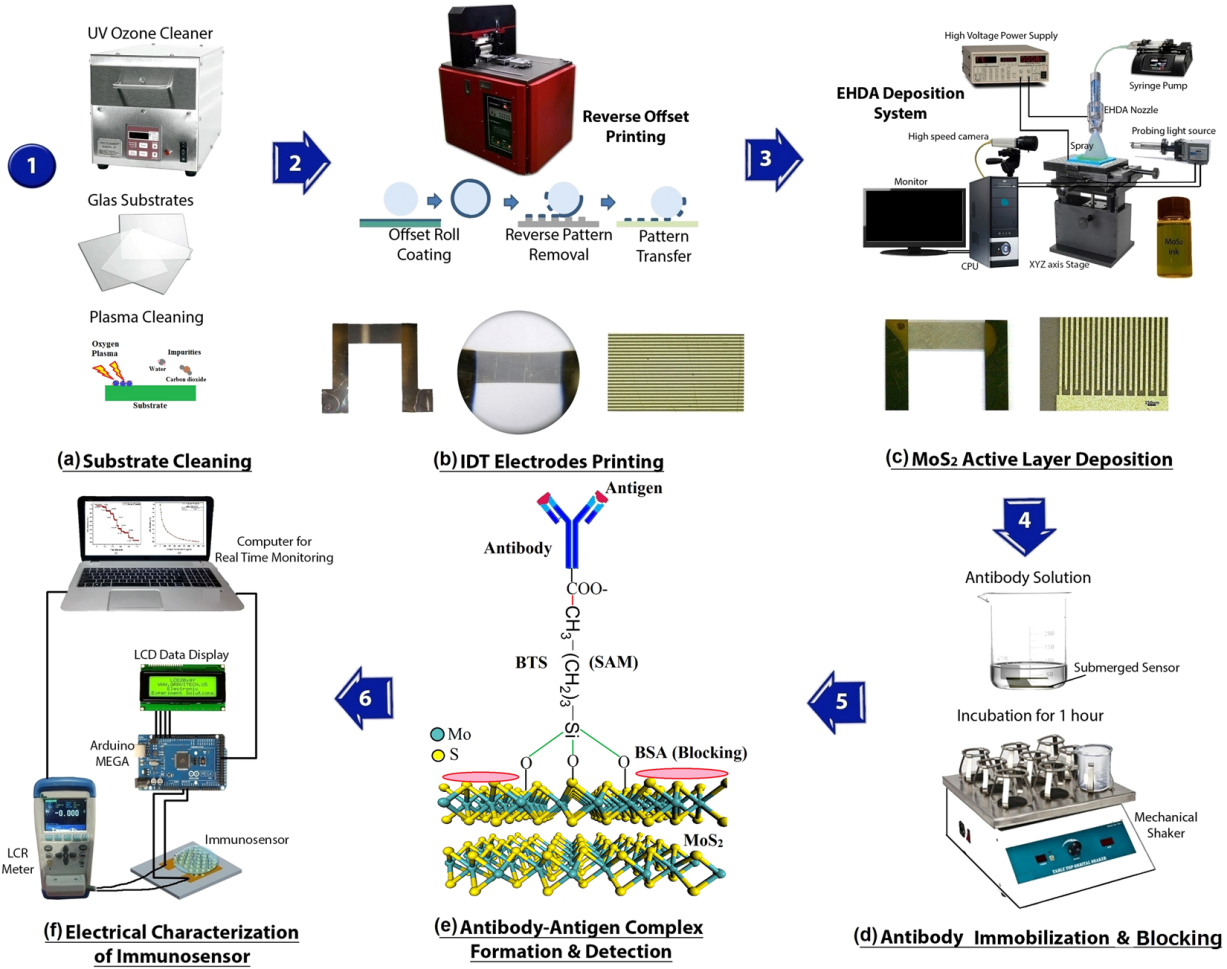
Process flow diagram of sensors fabrication and characterization. (**a**) Substrate cleaning by plasma, (**b**) Electrode fabrication by reverse offset printing, (**c**) MoS_2_ active layer deposition by EHDA, (**d**) Antibody immobilization and blocking, (**e**) Antibody-antigen complex, and (**f**) Sensors’ electrical response.

The step by step process flow diagram for the sensor fabrication is presented in Fig. 8. Glass was used as sensor substrate that was first cleaned and dried at room temperature. UV ozone plasma treatment for 5 minutes was used for the removal of any surface impurities and improving the hydrophilicity to facilitate the printing of electrodes^48^. Exactly identical IDT electrodes having 20 and 40 finger pairs with width of 50 µm were printed using reverse-offset printing system. The schematic of reverse offset printing is presented in Fig. 8(b).

### MoS_2_ exfoliation and deposition

Bulk MoS_2_ powder was dispersed in NMP solvent and was ground for 1 hour in a mortar to mechanically exfoliate the bigger chunks into smaller flakes. The resultant dispersion was then probe sonicated for 1 hour for further fine exfoliation of the MoS_2_ flakes. Liquid exfoliation was chosen instead of sticker based exfoliation to achieve few layered MoS_2_ flakes owing to the simpler process that can be used to achieve large quantities aiming at mass production. The remaining un-exfoliated bulk MoS_2_ flakes were separated from the solution using centrifugation at 4000 rpm for 20 minutes^31^. The resultant clear MoS_2_ solution ready for deposition has been shown in Fig. 8(c). MoS_2_ ink was then deposited on to the active area using Electrohydrodynamic Atomization (EHDA) process. EHDA was used owing to its high accuracy, excellent thin film fabrication characteristics, and compatibility with mass production. A schematic diagram for EHDA has been presented in Fig. 8(c). The major parameters for EHDA deposition include applied voltage, ink flow rate, and nozzle to substrate distance. The parameters were optimized for MoS_2_ film fabrication following the method presented in our previous work^49^. The images of stable jet and the table of optimized parameters are presented in Supplementary Figure S1 and Table S1 respectively. After MoS_2_ deposition, the sensors were cured at 120 °C for 2 hours to evaporate any traces of solvent left in the active layer of MoS_2_. The surface of the few layered 2D sheets were then functionalized through silane based method using 2 vol% butyl-trichlorosilanes BTS^50–55^. This step is eminent to improve the specificity and reduce the non-specific absorption^56^.

### Equipment

The sensors were characterized for their chemical and physical properties using field emission scanning electron microscopy (FE-SEM), Atomic Force Microscopy (AFM), Raman spectroscopy, X-ray photoelectron spectroscopy (XPS), photoluminescence (PL) emission spectra, and laser confocal fluorescent microscopy. Raman spectra was recorded on (LabRAM HR Raman) spectrometer with a solid state laser at room temperature with the excitation laser wavelength of 514 nm. Photoluminescence (PL) spectra were recorded by a (LS-55 Perkin Elmer Fluorescence Spectrometer) ranging from 350 nm to 700 nm. Carl Zeiss Supra 55VP was used for SEM. PHI Quantera II VG X-ray photoelectron spectroscopy was used for XPS. Olympus FV500 confocal microscope was used for laser fluorescent microscopy. Electrical characterizations were carried out using Applent AT-826 LCR meter with 0.6Vrms sinusoidal output.

## Electronic supplementary material

Video S1

Supplementary Information

## References

[1] Carlos Moina, G. Y. Fundamentals and applications of Immunosensors. *Adv*. *Immunoass*. *Technol*. 111, doi:10.1002/1521-3773 (2012).

[2] Luppa PB (2001). Immunosensors—principles and applications to clinical chemistry. Clin. Chim. Acta.

[3] Gerwen PV (1998). Nanoscaled interdigitated electrode arrays for biochemical sensors. Sensors Actuators B Chem..

[4] Duan D (2015). Nanozyme-strip for rapid local diagnosis of Ebola. Biosens. Bioelectron..

[5] Nidzworski D (2014). Universal biosensor for detection of influenza virus. Biosens. Bioelectron..

[6] Andreescu. Trends and challenges in biochemical sensors for clinical and environmental monitoring*. *Pure Appl*. *Chem* **76**, 861–878 (2004).

[7] Shavanova K (2016). Application of 2D non-graphene materials and 2D oxide nanostructures for biosensing technology. Sensors (Switzerland).

[8] Varghese S (2015). Two-Dimensional Materials for Sensing: Graphene and Beyond. Electronics.

[9] Chao CY, Guo LJ (2003). Biochemical sensors based on polymer microrings with sharp asymmetrical resonance. Appl. Phys. Lett..

[10] Barbosa AI, Gehlot P, Sidapra K, Edwards AD, Reis NM (2015). Portable smartphone quantitation of prostate specific antigen (PSA) in a fluoropolymer microfluidic device. Biosens. Bioelectron..

[11] Quesada-González D, Merkoçi A (2015). Nanoparticle-based lateral flow biosensors. Biosens. Bioelectron..

[12] Zhu, C. Electrochemical Sensors and Biosensors Based on Nanomaterials and Nanostructures. *Anal*. *Chem*., doi:10.1021/ac5039863 (2014). 10.1021/ac5039863 PMC4287168 25354297

[13] Daniels. Label-free impedance biosensors: Opportunities and challenges. *Electroanalysis* **19**, 1239–1257 (2007). 10.1002/elan.200603855 PMC2174792 18176631

[14] Kumar S (2015). Reduced graphene oxide modified smart conducting paper for cancer biosensor. Biosens. Bioelectron..

[15] Liu, Y., Xie, Y. & Feng, C. Potentiometric immunosensor with a novel immobilization procedure based on a nano-Au-modified planar gold electrode. In *5th International Conference on Bioinformatics and Biomedical Engineering*, *iCBBE 2011* 4–7, doi:10.1109/icbbe.2011.5780309 (2011).

[16] Sharma, M. K. Highly Sensitive Amperometric Immunosensor for Detection of Plasmodium falciparum Histidine-Rich Protein 2 in Serum of Humans with Malaria: Comparison with a Commercial Kit Highly Sensitive Amperometric Immunosensor for Detection of Plasmodium falciparum. *J*. *Clin*. *Microbiol*. 1–8, doi:10.1128/JCM.01022-08 (2008). 10.1128/JCM.01022-08 PMC2576608 18799699

[17] Lee CS (2010). A highly sensitive enzyme-amplified immunosensor based on a nanoporous niobium oxide (Nb2O5) electrode. Sensors.

[18] Mannoor MS (2012). Graphene-based wireless bacteria detection on tooth enamel. Nat. Commun..

[19] Cramer T (2013). Water-gated organic field effect transistors – opportunities for biochemical sensing and extracellular signal transduction. J. Mater. Chem. B.

[20] Lee J (2014). Two-dimensional layered MoS_2_ biosensors enable highly sensitive detection of biomolecules. Sci. Rep..

[21] Sarkar D (2014). MoS2 field-effect transistor for next-generation label-free biosensors. ACS Nano.

[22] Wang QH (2012). Electronics and optoelectronics of two-dimensional transition metal dichalcogenides. Nat. Nanotechnol..

[23] Nam H (2015). Fabrication and comparison of MoS2 and WSe2 field-effect transistor biosensors. J. Vac. Sci. Technol. B, Nanotechnol. Microelectron. Mater. Process. Meas. Phenom..

[24] Li H (2012). Fabrication of single- and multilayer MoS_2_ film-based field-effect transistors for sensing NO at room temperature. Small.

[25] Perkins, F. K. *et al*. Chemical Vapor Sensing with Monolayer MoS_2_ (2013). 10.1021/nl3043079 23339527

[26] Sekretaryova AN (2015). Bioelectrocatalytic systems for health applications. Biotechnol. Adv..

[27] Dhruv, H. D. Controlling Nonspecific Adsorption of Proteins at Bio-Interfaces for Biosensor and Biomedical Applications. *Merrill cazier library* (Utah State University). doi:Paper-276 (2009).

[28] Chin, C. D. *Low-Cost Microdevices for Point-of-Care Testing*, doi:10.1007/978-3-642-29268-2 (2013).

[29] Wang, F. An Organ-Like Titanium Carbide Material (MXene) with Multilayer Structure Encapsulating Hemoglobin for a Mediator-Free Biosensor. **162**, 16–21 (2015).

[30] Choi S, Chae J (2010). Methods of reducing non-specific adsorption in microfluidic biosensors. J. Micromechanics Microengineering.

[31] Rehman MM (2016). Resistive Switching in All-Printed, Flexible and Hybrid MoS2-PVA Nanocomposite based Memristive Device Fabricated by Reverse Offset. Sci. Rep..

[32] Ali J, Uddin G, Hyun K, Jang Y, Lee K (2016). Fabrication of blue luminescent MoS_2_ quantum dots by wet grinding assisted co-solvent sonication. J. Lumin..

[33] Kavosi B (2015). Ultrasensitive electrochemical immunosensor for PSA biomarker detection in prostate cancer cells using gold nanoparticles/PAMAM dendrimer loaded with enzyme linked aptamer as integrated triple signal ampli fi cation strateg. Biosens. Bioelectron..

[34] Wu D (2016). Label-free Electrochemiluminescent Immunosensor for Detection of Prostate Specific Antigen based on Aminated Graphene Quantum Dots and Carboxyl Graphene Quantum Dots. Sci. Rep..

[35] Wang, Y. Fabrication of Relative Humidity Sensors based on Polyimide Nanoparticles. *Thesis SIMON FRASER Univ*. (2013).

[36] Loeb S, Catalona WJ (2014). The Prostate Health Index: a new test for the detection of prostate cancer. Ther. Adv. Urol..

[37] Azzouzi S (2016). Sensors and Actuators B: Chemical Citrate-selective electrochemical -sensor for early stage detection of prostate cancer. Sensors Actuators B. Chem..

[38] Gonzalez-Quintela A (2008). Serum levels of immunoglobulins (IgG, IgA, IgM) in a general adult population and their relationship with alcohol consumption, smoking and common metabolic abnormalities. Clin. Exp. Immunol..

[39] Wang J (2007). Immunosensors Based on Functional Nanoparticle Labels. ECS Trans..

[40] Das R (2015). Controllable gold nanoparticle deposition on carbon nanotubes and their application in immunosensing. RSC Adv..

[41] Aziz, A. & Yang, H. Electrochemical Immunosensor Using the Modification of an Amine-functionalized Indium Tin Oxide Electrode with Carboxylated Single-walled Carbon Nanotubes. **28**, 1171–1174 (2007).

[42] Wu Y (2009). A novel reagentless amperometric immunosensor based on gold nanoparticles/TMB/Nafion-modified electrode. Biosens. Bioelectron..

[43] Xu HY, Aguilar ZP, Wang AY (2010). Quantum Dot-based Sensors for Proteins. ECS Trans..

[44] Minamiki T (2014). A label-free immunosensor for IgG based on an extended-gate type organic field effect transistor. Materials (Basel)..

[45] Baldini F (2008). FRET based biosensor for detection of active NF-kB. DNA Seq..

[46] Oeckinghaus A, Ghosh S (2009). The NF-kappaB family of transcription factors and its regulation. Cold Spring Harbor perspectives in biology.

[47] Shen Q (2014). Adipocyte reporter assays: Application for identification of anti-inflammatory and antioxidant properties of mangosteen xanthones. Mol. Nutr. Food Res..

[48] Sajid M (2016). Bio-compatible organic humidity sensor transferred to arbitrary surfaces fabricated using single-cell-thick onion membrane as both the substrate and sensing layer. Sci. Rep..

[49] Choi KH, Siddiqui GU, Yang B, Mustafa M (2015). Synthesis of ZnSnO3 nanocubes and thin film fabrication of (ZnSnO3/PMMA) composite through electrospray deposition. J. Mater. Sci. Mater. Electron..

[50] Wang L (2014). Functionalized MoS2 nanosheet-based field-effect biosensor for label-free sensitive detection of cancer marker proteins in solution. Small.

[51] Chua JH, Chee RE, Agarwal A, She MW, Zhang GJ (2009). Label-free electrical detection of cardiac biomarker with complementary metal-oxide semiconductor-compatible silicon nanowire sensor arrays. Anal. Chem..

[52] Patolsky F, Zheng G, Lieber CM (2006). Fabrication of silicon nanowire devices for ultrasensitive, label-free, real-time detection of biological and chemical species. Nat. Protoc..

[53] Kim A (2007). Ultrasensitive, label-free, and real-time immunodetection using silicon field-effect transistors. Appl. Phys. Lett..

[54] Zheng G, Patolsky F, Cui Y, Wang WU, Lieber CM (2005). Multiplexed electrical detection of cancer markers with nanowire sensor arrays. Nat. Biotechnol..

[55] Lin MC (2007). Control and detection of organosilane polarization on nanowire field-effect transistors. Nano Lett..

[56] Kalantar-zadeh K, Ou JZ (2016). Biosensors Based on Two-Dimensional MoS_2_. ACS Sensors.

