# Supplementary material for: All-printed highly sensitive 2D MoS2 based multi-reagent immunosensor for smartphone based point-of-care diagnosis
Source: Sci Rep. 2017 Jul 19;7:5802. doi: 10.1038/s41598-017-06265-1 (PMC5517636; doi:10.1038/s41598-017-06265-1)
Supplement: Supplementary file 2 — Supplementary Information [file 41598_2017_6265_MOESM2_ESM.pdf]

Supplementary Information

All-printed highly sensitive 2D MoS_2 based multi-reagent immunosensor for smartphone based point-of-care diagnosis

Memoon Sajid [1†], Ahmed Osman [2, 3†], Ghayas Uddin Siddiqui [1], Hyun Bum Kim [1], Soo Wan Kim [1], Jeong Bum Ko [1], Yoon Kyu Lim [4], Kyung Hyun Choi [1,]*

Electrohydrodynamic Atomization Spray

The multiple modes of spray cone jet for EHDA deposition process are presented in Fig. S1 below. The figure indicates that the first mode is dripping mode where the materials droplets are ejected without any cone jet formation. The second mode is micro-dripping mode where the ejected droplet size is reduced but still there is no formation of cone jet. The third mode is stable cone jet mode while further increasing the voltage will lead to unstable and multi-jet modes.

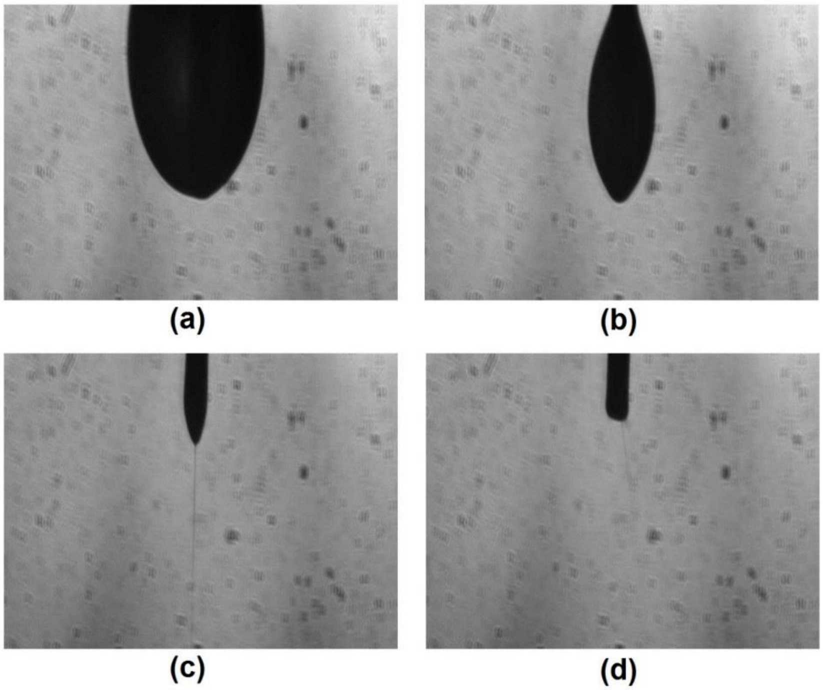

Figure S1 | Different modes of EHDA printing (a) dripping, **(b)** micro-dripping, **(c)** stable cone jet, and **(d)** unstable multi-jet

The system parameters are optimized to achieve a stable cone jet mode for the thin film deposition of MoS_2. The system parameters are presented in Table S1.

Table S1 | Optimized system parameters for the deposition of MoS2 thin film using EHDA technique

Parameter	Optimized Value
Flow Rate	200 µl/hr.
Nozzle to Substrate Distance	15 mm
Stage Speed	5 mm/s
Nozzle Diameter	210 µm
Number of Passes	5
Voltage Range for Stable Jet	5.4-6.1 kV
Syringe Internal Diameter	12 mm

The film thickness of the deposited MoS_2 flakes was measured using cross-sectional SEM image presented in Fig. S2 and was found to be ~130 nm.

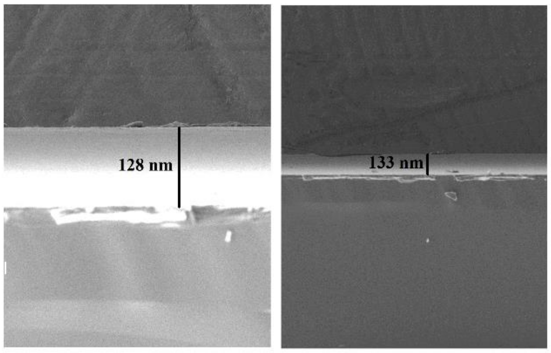

Figure S2 | Cross-sectional SEM image of functionalized MoS_2 showing film thickness

Surface functionalization

The surface functionalization method and working principle is based on already reported methods in the literature with little modifications [1–6]. As a brief summary, the MoS_2 thin films submerged in the solution of ethanol and water (90% and 10% respectively) for 1 hour to produce free hydroxyl (OH) groups on MoS_2 surface [1,5]. Afterwards, for surface functionalization, BTS was added drop by drop in the solution at 2% vol/vol during continuous

mechanical shaking. The Si atoms of BTS are attached to the MoS_2 surface via silane bonding. The samples were gently washed by IPA, dried with N_2, and were then heated at 110ºC for 60 min. After functionalization, the sensors were submerged in antibody solution containing BSA as the blocking agent and were incubated for antibody attachment to the SAMs. The FTIR spectrum of functionalized MoS_2 has been shown in Fig. S3. The absorption bands at 2343 and 2359 cm^{-1} may correspond to silane bonding [7]. The absorption peaks at 2873 and 2927 cm^{-1} correspond to asymmetric and symmetric stretching vibrations of methylene (CH_2) group whereas the absorption peak at 2957 cm^{-1} is attributed to the methyl (CH_3) stretching vibration [8,9]. This triplet band confirms the presence of alkyl-silane bonding on the MoS_2 surface. The absorption bands at 1377 and 1408 cm^{-1} may be assigned to CH_3 deformation and C-O-H bending. The absorption peaks at 1202, 1274, 1302 and 1466 cm^{-1} are assigned to C-C stretching, C-O stretching, out of plane C-H bending and C-H bending (scissoring) respectively that can be attributed to antibody attachment to the silane. The peak at 992 cm^{-1} can be assigned to the Mo-S band [8]. The absorption peak at 886 cm^{-1} may be assigned to the Si-O group, while the absorption band at 769 cm^{-1} is assigned to Si-C group showing interaction between MoS_2 and silane.

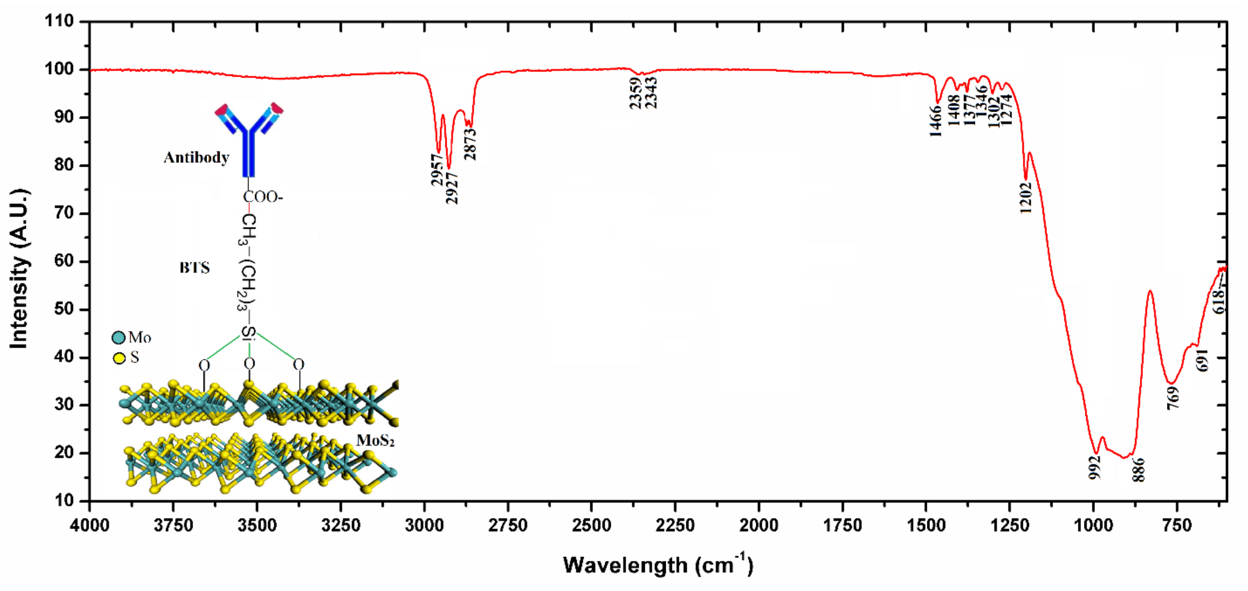

Figure S3 | FTIR spectrum of functionalized MoS_2 and the schematic diagram showing attached BTS SAM's and antibodies on to the surface.

In the functionalized thin films, the silane molecules displace the adsorbed water or OH groups from the MoS_2 surface. Because the water molecules tend to absorb on the defects rather than the perfect surface, it is clear that the silane moieties displace the water molecules from these defects [7,10]. These defects serve as the anchoring points (chemisorption) from which the polymerized BTS molecules can extend and cover larger areas of 2D MoS_2 flakes (physisorption) [7,10]. This method almost ensures that even if the defects are not perfectly similar in the flakes, the resulting functionalization will be repeatable [11] due to covering large area of the flakes with BTS and the sensor results for multiple trials and samples with little error prove the repeatability.

Point of care diagnosis system and process

The basic circuit diagram for the detection circuit is presented here in Fig. S4 (A) but the detailed circuit diagram is not presented that also includes amplification and conditioning portions to improve signal to noise ratio (SNR) and sensitivity. First, the reading of the current without the control solvent was recorded and then the control was added to start calibration and record the base impedance. After that, a known amount of antigen is added to the control solvent and the system automatically waits for the readings to get stabilized. The percentage sensitivity is calculated and stored.

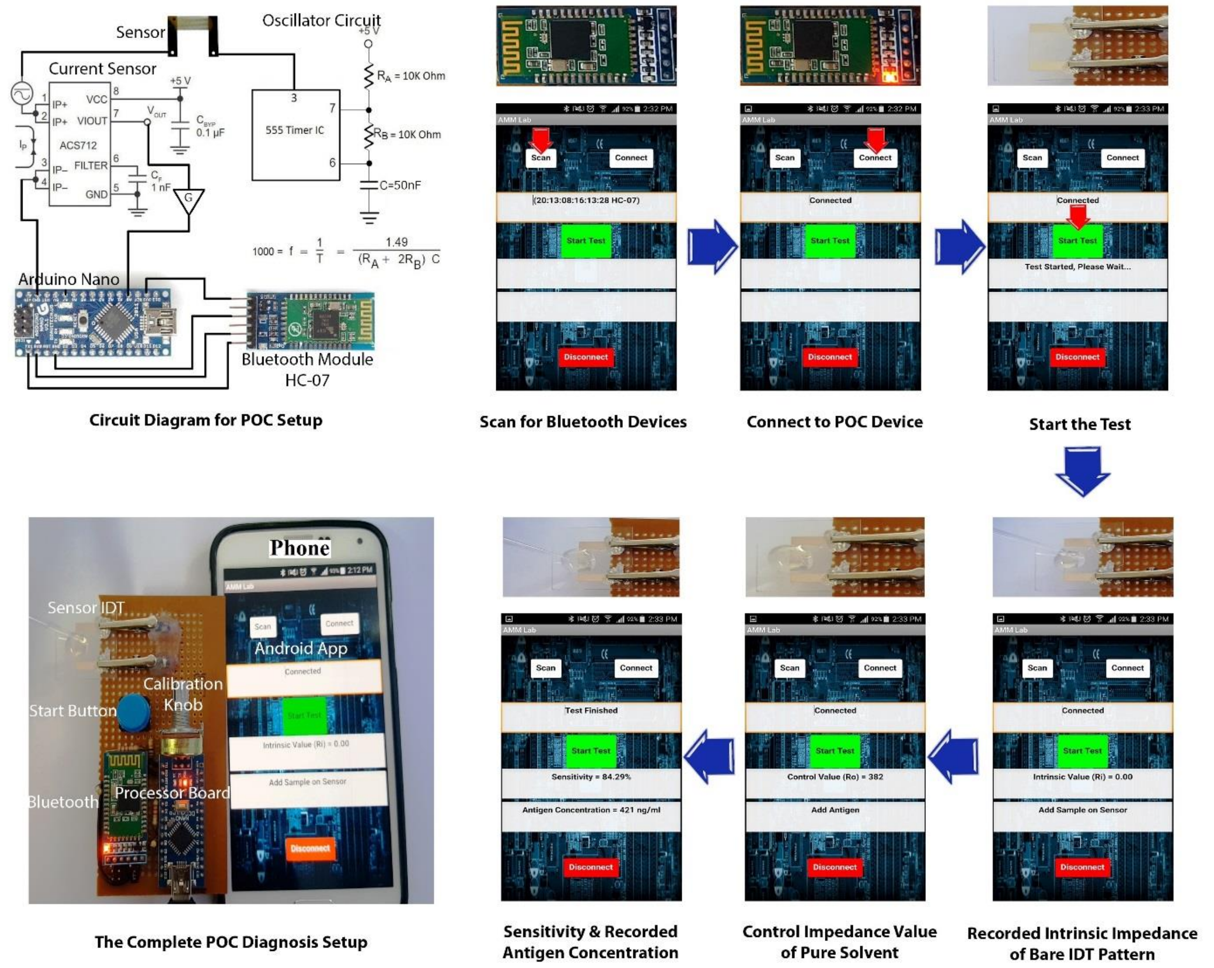

Figure S4 | Point of Care (POC) Diagnosis process with (a) showing the circuit diagram, **(b)** showing the real picture of setup, and **(c)** showing the step by step diagnosis process using the developed Android smart phone application

References

1. Wang, L. *et al.* Functionalized MoS2 nanosheet-based field-effect biosensor for label-free sensitive detection of cancer marker proteins in solution. *Small* **10,** 1101–1105 (2014).
2. Chua, J. H., Chee, R. E., Agarwal, A., She, M. W. & Zhang, G. J. Label-free electrical detection of cardiac biomarker with complementary metal-oxide semiconductor-compatible silicon nanowire sensor arrays. *Anal. Chem.* **81,** 6266–6271 (2009).
3. Patolsky, F., Zheng, G. & Lieber, C. M. Fabrication of silicon nanowire devices for ultrasensitive, label-free, real-time detection of biological and chemical species. *Nat. Protoc.* **1,** 1711–1724 (2006).
4. Kim, A. *et al.* Ultrasensitive, label-free, and real-time immunodetection using silicon field-effect transistors. *Appl. Phys. Lett.* **91,** 11–14 (2007).
5. Zheng, G., Patolsky, F., Cui, Y., Wang, W. U. & Lieber, C. M. Multiplexed electrical detection of cancer markers with nanowire sensor arrays. *Nat. Biotechnol.* **23,** 1294–1301 (2005).

6. Lin, M. C. *et al.* Control and detection of organosilane polarization on nanowire field-effect transistors. *Nano Lett.* **7,** 3656–3661 (2007).
7. Yu, X., Pr??vot, M. S. & Sivula, K. Multiflake thin film electronic devices of solution processed 2D MoS2 enabled by sonopolymer assisted exfoliation and surface modification. *Chem. Mater.* **26,** 5892–5899 (2014).
8. Liu, J., Li, Y., Ke, J., Wang, Z. & Xiao, H. Synergically Improving Light Harvesting and Charge Transportation of TiO2 Nanobelts by Deposition of MoS2 for Enhanced Photocatalytic Removal of Cr(VI). *Catalysts* **7,** 30 (2017).
9. Zeng, X. *et al.* Effect of Polymer Addition on the Structure and Hydrogen Evolution Reaction Property of Nanoflower-Like Molybdenum Disulfide. *Metals (Basel).* **5,** 1829–1844 (2015).
10. Shahar, C. *et al.* Surface functionalization of WS2 fullerene-like nanoparticles. *Langmuir* **26,** 4409–4414 (2010).
11. Sarkar, D. *et al.* MoS2 field-effect transistor for next-generation label-free biosensors. *ACS Nano* **8,** 3992–4003 (2014).
